# The Story of the Insane from Year to Year

**Published:** 1893-08-19

**Authors:** 


### THE STORY OF THE INSANE FROM YEAR TO YEAR.

Fifth Series.

### LEICESTER AND RUTLAND COUNTY ASYLUM.

In this asylum there was a decrease in the number resident from 471 to 453, this decrease being apparently caused by a rearrangement of the boundaries of the borough of Leicester, whereby certain districts formerly in the county are now included in the borough. One hundred and fifteen patients were admitted during the year: 40 were discharged recovered and 63 died. The percentage of recoveries stands at 35·71 and that of the deaths at 14. Intemperance in drink caused the insanity in 9 of the admissions and hereditary tendency in 23. These are both lower than the average, which seems the more remarkable as only one case is put down as unascertained; but 38 are stated to have been owing to "combined causes," which term probably includes both drink and hereditary. Of the deaths 15 were due to cerebral diseases, 8 to consumption, and 4 to diarrhœa. Dr. Higgins explains the somewhat high death-rate by drawing "attention to the unfavourable nature of a large proportion of the admissions last year, and especially to the feeble, aged, and diseased condition of many of the cases received. . . . Eleven of those admitted in 1891 died in a short time, 7 with an average of only ten days' residence and 4 within three months." The chaplain says that "one of Her Majesty's Commissioners made some strong observations last March on the sort of books supplied to the patients. The house visitors consequently inspected the library, and considered the remarks uncalled for." The weekly rate of maintenance was 9s. 7⅝d.

THE SALOP AND MONTGOMERY ASYLUM.

On the first day of January, 1891, there were 720 patients in the asylum, and by the end of the year the numbers had risen to 740, 25 of these being private cases. The admissions were 183, the recoveries were 63, and the deaths 68. The percentage of recoveries on admissions was 35·39, and that of the deaths on the average number resident was 9·28. As to the causes of insanity, hereditary predisposition, alone or combined with other causes, heads the list with 50 cases, and alcoholic intemperance accounts for 12. Forty-three of the deaths were due to brain diseases and seven to phthisis. The following case shows how determined lunatics are when they wish to commit suicide, and how impossible it is to forsee the means they will use to effect their purpose. This patient "was placed in a padded room in the infirmary ward. Although he was constantly visited he managed, with an amount of cleverness of a kind only to be found in a lunatic, to hang himself by putting his head through the sash cord of the shutter to the window of his room." The increase of work thrown on the clerks' department by the new Lunacy Act necessitated the appointment of an assistant clerk. Regarding the Act itself, Dr. Strange says: "Many superintendents have already expressed strong opinions on the latest addition to the Lunacy Acts. I have hitherto refrained from doing so, hoping that time would show some good in all the extra work thrown on asylum officials, and prove that the very elaborate precautions taken had been of some benefit to the insane. I can only say that up to this time there is no criticism that I have seen but which has been amply justified." The profits from the farm are stated to be £894. The weekly rate of maintenance is a fraction under 8s.

THE WEXFORD DISTRICT ASYLUM.

Like the most of the Irish asylums, the Enniscorthy Asylum is a small one. It contained 381 patients at the end of the year, and the increase during the year was almost nominal. There were 80 admissions, 39 recoveries, and 17 deaths. The percentage of recoveries was 48·75, and that of the deaths 4·41, both calculations being made in the manner usual in asylums. The recoveries are considerably above our English average, and the death-rate much below it. On looking over Table VIII., which gives the causes of death, we notice a total absence of general paralysis of the insane—a pecu-liarity by no means confined to Wexford, for it is, for some reason or other, very uncommon in Irish asylums. On the other hand, consumption accounted for five out of the 17 deaths. Intemperance in drink and hereditary influence are responsible for the insanity in 32 of the admissions. Dr. Drapes is not ashamed to record that he used mechanical restraint during the year, and we think he is quite justified in its use. He believes it to be preferable to seclusion in some cases of violence, and occasionally in those cases of semi-imbecility "with whom reasoning has no effect, and whose chief pleasure in life seems to consist in objectlessly tearing to shreds everything they can lay their hands on." We do not see any report from the Visiting Committee or from the Lunacy Inspectors. The farm is said to have yielded a profit of £218, but we see only £9 13s. charged for rent, and no interest on capital is put down, nor are the prices given of farm produce supplied to the asylum. The cost of maintenance per head per annum is £26 11s. 6d.

THE FIFE AND KINROSS ASYLUM.

This asylum contains about 440 patients. During the year 119 patients were admitted, 28 were discharged recovered, and 31 died. The recoveries stand at 30·17 per cent. and the deaths at 8·66. Twenty-six of the admissions are supposed to have been driven insane by drink, and hereditary influence accounts for 42. The asylum contains eighty patients in excess of what it has proper accommodation for, but with the opening of the new hospital this crowding will disappear and the asylum will again be brought "abreast of the demands made on it by the district." Dr. Turnbull has begun training classes for the attendants and nurses; and sixteen have been successful in passing these examinations. The weekly rate of maintenance is 9s. 1½d. per head per week.

